# Sleep and Tibialis Anterior Muscle Activity in Mice With Mild Hypoxia and Iron Deficiency: Implications for the Restless Legs Syndrome

**DOI:** 10.3389/fphys.2018.01818

**Published:** 2018-12-17

**Authors:** Viviana Lo Martire, Sara Alvente, Stefano Bastianini, Chiara Berteotti, Alice Valli, Mauro Manconi, Giovanna Zoccoli, Alessandro Silvani

**Affiliations:** ^1^Laboratory of Physiological Regulations in Sleeping Mice, Department of Biomedical and Neuromotor Sciences, University of Bologna, Bologna, Italy; ^2^Sleep and Epilepsy Center, Neurocenter of Southern Switzerland, Civic Hospital (EOC) of Lugano, Lugano, Switzerland; ^3^Department of Neurology, Bern University Hospital, Bern, Switzerland

**Keywords:** hypoxia, iron, mice, restless legs syndrome, Willis-Ekbom disease, sleep, circadian, periodic leg movements during sleep

## Abstract

Restless legs syndrome (RLS) is a neurological disorder that entails an urge to move with a circadian pattern during the evening/night. RLS may be accompanied by decreased sleep time and increased occurrence of periodic leg movements during sleep (PLMS), which involve bursts of tibialis anterior (TA) muscle electromyogram (EMG). Mild hypoxia and non-anemic iron deficiency, a highly prevalent nutritional deficiency, are relatively unexplored factors in RLS pathophysiology. We tested whether mice exposed to mild hypoxia, alone or in combination with non-anemic iron deficiency, show decreased sleep time particularly in the light (rest) period and increased occurrence of TA EMG phasic events similar to human PLMS. Female C57BL/6J mice were fed diets with low or normal iron for 6 months from weaning and instrumented with electrodes to record the electroencephalogram and the EMG of both TA muscles. Mice were recorded in a whole-body plethysmograph while breathing a normoxic or mildly hypoxic (15% O_2_) gas mixture for 48 h. Hypoxia increased minute ventilation during sleep. The low-iron diet decreased liver and serum iron, leaving blood hemoglobin and brainstem iron levels unaffected. Hypoxia, either alone or in combination with non-anemic iron deficiency, decreased non-rapid-eye-movement (non-REM) sleep time, but this occurred irrespective of the light/dark period and was not associated with increased occurrence of TA EMG events during non-REM sleep. These results do not support the hypothesis that mild hypoxia is sufficient to cause signs of RLS, either alone or in combination with non-anemic iron deficiency, pointing to the necessity of further susceptibility factors.

## Introduction

Restless legs syndrome (RLS) is a chronic neurological disorder that entails the irresistible urge to move the legs, which occurs after a period of inactivity and improves or disappears with movement (Wijemanne and Ondo, 2017). The symptoms follow a circadian trend, developing or worsening in the evening or at night (Hening et al., 1999; Michaud et al., 2005). RLS also typically entails objective signs, which include disturbed nocturnal sleep with decreased sleep time (Hornyak et al., 2007) and periodic limb movements during sleep (PLMS) involving repetitive series of phasic increases in tibialis anterior (TA) electromyogram (EMG) (Montplaisir et al., 1997). The prevalence of RLS is estimated at 1.9–4.6% with the strictest criteria, and is about twice as high in women than in men (Ohayon et al., 2012). RLS decreases the quality of life to a level similar to other chronic diseases such as diabetes type 2, depression, and osteoarthritis (Stevens, 2015). Despite the availability of multiple drug options, long-term treatment management is often difficult particularly because of augmentation, a paradoxical increase in symptom severity in the course of dopamine-agonist therapy (Garcia-Borreguero et al., 2018). This highlights the unmet need for better long-term therapies for RLS, which is at least partly due to the uncertainty that still surrounds RLS pathophysiology.

The relationship between RLS and hypoxia is one of the most recent and interesting hypotheses on RLS pathophysiology. An almost threefold prevalence of RLS occurs in patients with chronic obstructive pulmonary disease compared to age- and sex-matched control subjects (Lo Coco et al., 2009), and symptoms of RLS are more prevalent among women at high risk for obstructive sleep apnea than in women at low risk (Kapsimalis and Kryger, 2009). On the other hand, patients with RLS show greater capillary tortuosity in TA muscles than control subjects, suggesting a role of hypoxia as a factor causing capillary remodeling (Larsson et al., 2007). Accordingly, transcutaneous oxygen pressure is lower in patients with RLS than in healthy subjects in the legs, but not in the trunk (Salminen et al., 2014). In different geographic areas, RLS prevalence increases in subjects living at 1800–2800 m above sea level (Sevim et al., 2003; Castillo et al., 2006; Gupta et al., 2017). At these altitudes, the low barometric pressure may cause mild hypoxic hypoxia comparable to that experienced by passengers on commercial aircraft flights, or to that caused by reducing the inspired O_2_ fraction from 21 to 15% at sea level (Nicholson and Sznajder, 2014). It is still unclear whether such mild levels of hypoxia may be causal in triggering RLS, either *per se* or in combination with other susceptibility factors.

Different lines of evidence suggest that iron deficiency is a susceptibility factor for RLS, possibly in connection with changes in mitochondrial biogenesis and/or in the expression of HIF, the hypoxia inducible factor (Earley et al., 2014; Connor et al., 2017). Conditions of iron deficiency severe enough to cause anemia are strongly associated with RLS (Trenkwalder et al., 2016), but do not account for the large prevalence of RLS in the general population. Milder conditions of non-anemic iron deficiency are estimated to be much more prevalent (Zimmermann and Hurrell, 2007), but their clinical relevance in general and for RLS in particular is still poorly understood. Nonetheless, non-anemic iron deficiency causes symptoms of fatigue that are lessened by iron supplementation (Houston et al., 2018), and exaggerates pulmonary vasoconstriction in conditions of mild hypoxia (Frise et al., 2016). Non-anemic iron deficiency may thus represent a highly prevalent susceptibility factor for RLS, particularly in combination with mild hypoxia.

Here, we aimed for a proof of principle that mild hypoxia plays a causal role in promoting RLS, either *per se* or in combination with non-anemic iron deficiency. We performed our experiments on an animal model (female C57BL/6J mice) in order to achieve the full experimental control needed for causal inferences, and because establishing an animal model would accelerate the search of novel druggable pathways of RLS pathophysiology. RLS diagnosis is based on subjective symptoms (Wijemanne and Ondo, 2017), which are hardly amenable to translation on animal models. We thus focused on the objective signs in mice that would be consistent with those commonly associated with RLS in patients: a reduction in sleep time with a characteristic circadian pattern of prevalence during the light (rest) period and an increased occurrence of phasic TA EMG events during sleep, with features similar to those of human PLMS (Montplaisir et al., 1997; Hening et al., 1999; Michaud et al., 2005; Hornyak et al., 2007).

## Materials and Methods

### Ethics Statement

The study protocol complied with the EU Directive 2010/63/EU for animal experiments and with Italian law (DL 26, March 4, 2014) and was approved by the animal welfare committee at the University of Bologna, Italy, and by the Italian Ministry of Health (protocol n. 245/2015-PR). All efforts were made to minimize animal suffering.

### Mice

Experiments were performed on 22 female wild-type mice of the C57BL/6J strain. Mice were bred from a colony expanded at the Department of Biomedical and Neuromotor Sciences of the University of Bologna, Italy, from founder mice bought from Charles River Italy (Calco, Italy). Mice were housed under a 12:12-h light–dark cycle with lights on (i.e., Zeitgeber Time 0, ZT 0) at 9 am, ambient temperature set at 21–23°C, and free access to water and food. Breeders were administered a standard rodent diet (4RF21; Mucedola, Settimo Milanese, Italy).

### Dietary Treatment

At weaning, which occurred at 3.7 ± 0.1 weeks of age, female mice were randomly allocated into two groups, which were fed either a low-iron rodent diet (iron deficient group, ID; EF/RM pelleted diet with <9 ppm iron; ssniff Spezialdiäten GmbH, Soest, Germany) or a nutrient-balanced control diet with iron supplementation (control group, Ctrl; E15510-04 diet with 179 ppm iron; ssniff Spezialdiäten GmbH). Thereafter, each diet was provided *ad libitum* until the termination of the experimental protocol, which occurred after 24.1 ± 0.6 weeks at euthanasia after the completion of recordings.

### Surgery

Mice underwent surgery at the age of 25–26 weeks for the implantation of screw electrodes to record the electroencephalogram (EEG, fronto-parietal differential lead) and of a couple of wire electrodes in each TA muscle to record the EMG, as previously described in detail (Silvani et al., 2015). The free ends of the TA EMG electrodes were tunneled subcutaneously to the mouse head. All electrodes were connected to a miniature custom-built socket (701-9925 RS Components, Cinisello Balsamo, Italy), which was cemented to the skull with stainless-steel anchor screws (Plastics One, Roanoke, VA, United States), dental cement (Rely X ARC, 3M ESPE, Segrate, Italy), and dental acrylic (Respal NF, SPD, Mulazzano, Italy). At the end of surgery, mice were administered benzathine penicillin (3750 UI/mouse) and dihydrostreptomycin sulfate (1.5 mg/mouse) in 800 μL saline subcutaneously to prevent infections and dehydration. All surgical procedures were performed under isoflurane anesthesia (1.8–2.4% in O_2_, inhalation route) with intra-operative analgesia (Carprofen 0.1 mg subcutaneously, Pfizer Italy, Latina) on a heating pad to prevent mouse hypothermia. A 1 week’s recovery from surgery was allowed before the start of the recordings procedures.

### Experimental Protocol

Upon completion of postoperative recovery, mice were connected to a lightweight cable for transmission of EEG and TA EMG signals. The experimental schedule was performed on four consecutive days. On days 1 and 2, the mice were continuously exposed to a normoxic gas mixture (21% O_2_ in N_2_) during a 24 h period (day 1) of habituation to the experimental apparatus and a subsequent 24 h period (day 2) of recordings. Then, on days 3 and 4, the same procedures as in the previous 2 days were repeated with continuous exposure to a hypoxic gas mixture (15% O_2_ in N_2_). Transitions from the habituation to the recordings environment were performed at ZT 3.

At the end of the recording session in conditions of hypoxia, mice were euthanized by anesthetic overdose (isoflurane 4% in O_2_) for harvesting of blood, brainstem, and liver samples, and autopsied. Liver samples weighting 60–90 mg were dissected taking care to avoid including major blood vessels and the gallbladder. An aliquot of whole blood was heparinized to prevent clotting and stored at -80°C for subsequent analysis of hemoglobin concentration, together with the brain and liver samples. Another aliquot of blood was centrifuged at 3000 rpm for 15 min at ambient temperature to obtain the serum, which was stored at -80°C for subsequent measurement of iron concentration. Sterile test tubes were employed for sample storage.

### Recordings

Simultaneous recordings of EEG, bilateral TA EMG, and breathing were performed as previously described in detail (Silvani et al., 2014) inside a modified 2-chamber whole-body plethysmograph (PLY4223, Buxco, Wilmington, NC, United States) consisting of a mouse chamber and a reference chamber. The mouse chamber was modified to decrease its internal volume to 0.97 L and to house a rotating electrical swivel (SL6C/SB, Plastics One) to prevent twisting of the mouse wire tether, and probes to measure temperature and humidity (PC52-4-SX-T3 sensor, Rense Instruments, Rowley, MA, United States). The whole-body plethysmograph was calibrated dynamically with a 100 μL micro-syringe (Hamilton, Reno, NV, United States) at the termination of each recording session. Habituation was performed inside a dummy whole-body plethysmograph with the same base area and made of the same material as the actual plethysmograph, equipped with a rotating dummy swivel. The true and dummy plethysmographs were continuously flushed with 1.5 L/min of normoxic or hypoxic gas mixtures, depending on the experimental session, in order to prevent buildup of CO_2_ and humidity. Food (low-iron or control diet depending on the group) and water were always provided *ad libitum*.

The EEG and EMG signals were acquired via wire transmission, amplified, and filtered (EEG, 0.3–100 Hz; EMG, 10–1000 Hz) using 7P511J amplifiers (Grass, West Warwick, RI, United States). The differential pressure between the mouse chamber and the reference chamber was measured with a high-precision pressure transducer (DP103-06, Validyne Engineering, Northridge, CA, United States), and digitized, together with the signals of EEG, EMG, and mouse chamber temperature and humidity, at 16-bit and 1024 Hz with a PCI-6224 board (National Instruments, Austin, TX, United States) operated by software written in the laboratory using Labview (National Instruments).

### Sleep Scoring

The states of wakefulness, non-rapid-eye-movement (non-REM) sleep, and REM sleep were scored with a 4-s time resolution based on qualitative visual inspection of the EEG signal and of the frequency, amplitude, and baseline value of raw respiratory recordings, as previously described in detail (Bastianini et al., 2017). Briefly, wakefulness was scored when the EEG was at a low voltage and included mixed frequencies, and the baseline value of the respiratory signal was highly irregular due to gross body movements that caused pressure artifacts obscuring individual breaths. Non-REM sleep was scored when the EEG was at a high voltage with prominent δ (0.5–4 Hz) frequency components, breathing frequency and amplitude were stable and regular, and the baseline value of the respiratory signal was steady. REM sleep was scored when the EEG was at a low voltage with predominant 𝜃 (6–9 Hz) frequency components, breathing frequency and amplitude were irregular, and the baseline value of the respiratory signal was steady. Scoring of all tracings was performed by a single researcher (SB) highly trained in this procedure for maximal accuracy. The percentages of recording time spent in wakefulness, non-REM sleep and REM sleep were eventually averaged over 3-h time bin and analyzed as a function of hypoxia/normoxia, low/normal dietary iron, and ZT.

### Quantitative Analysis of Breathing During Non-REM Sleep and REM Sleep

The values of ventilatory period (i.e., the interval between successive breaths, T_TOT_), tidal volume (VT) per gram body weight, and minute ventilation (i.e., VT/T_TOT_) per gram body weight were computed on non-REM and REM sleep episodes lasting ≥12 s (i.e., at least 3 consecutive 4-s epochs) as previously described in detail (Silvani et al., 2014). Breaths were identified automatically from the upward (+) deflection peak of plethysmograph pressure. Errors in breath detection as well as pressure artifacts (e.g., due to opening and closing of the room door) were manually excluded from the analyses. The analysis was not performed during wakefulness, when, as mentioned above, the marked irregularity of respiratory signal baseline often obscured individual breaths. The values of minute ventilation, VT, and T_TOT_ were eventually averaged over all recorded episodes of non-REM sleep and REM sleep and analyzed as a function of hypoxia/normoxia, low/normal dietary iron, and sleep state.

### Quantitative Analysis of TA EMG Bursts During Non-REM Sleep

The TA EMG bursts were scored and analyzed with a two-step semi-automated procedure during non-REM sleep as previously described in detail (Silvani et al., 2015). In the present study, the isolated 4-s epochs scored as wakefulness that were preceded and followed by non-REM sleep epochs were treated as non-REM sleep for the purpose of the TA EMG burst analysis. This was meant to avoid excluding the largest brief TA EMG bursts during non-REM sleep, which may cause irregularities of the plethysmographic signal baseline scored as single epochs of wakefulness according to the criteria listed above.

The first step of the analysis of TA EMG bursts was performed with an algorithm for automatic scoring, which was previously validated against consensus visual scoring of TA EMG bursts in mice, with a sensitivity of 90% and a false positive rate of 31% (Silvani et al., 2015). The second step of TA EMG bursts analysis consisted of a manual editing of the results of the automatic scoring based on the raw tracings of TA EMG. This second step was aimed to detect all phasic motor events that involved one or both TA muscles, entailed EMG bursts clearly discernible against the EMG background, and had duration <4 s, following previously published scoring rules (Silvani et al., 2015). To maximize accuracy, the TA EMG bursts of all mice were visually scored by the same researcher highly trained in this procedure (VLM).

According to previous work (Silvani et al., 2015), the TA EMG bursts that overlapped to any extent were considered to represent the same TA EMG *event* regardless of whether they occurred in different limbs. The time structure of TA EMG events was evaluated by computing the intervals between the onset of consecutive events (inter-event intervals, IEI). The IEI distribution, i.e., the number of IEI scored per hour of non-REM sleep grouped in 5-s intervals up to 60 s, was computed as a function of hypoxia/normoxia, low/normal dietary iron, and light/dark period. The upper IEI limit of 60 s was chosen in agreement with a previous study on mice (Silvani et al., 2015) and corresponds to the recently proposed, data-driven upper limit for the inter-movement intervals of PLMS in patients with RLS (Ferri et al., 2018). The occurrence rate of four different categories of TA EMG events was compared as a function of hypoxia/normoxia, low/normal dietary iron, and sleep state: (1) all TA EMG events, regardless of their IEI; (2) short-interval TA EMG events with IEI between 0.5 and 10 s (Silvani et al., 2015); (3) long-interval TA EMG events with IEI between 10 and 60 s; (4) long-interval TA EMG events with IEI between 10 and 60 s organized in series of 4 or more events, which most closely correspond to human PLMS (Ferri et al., 2018). The 0.5 and 10 s boundaries of short-interval TA EMG events were chosen in agreement with previous work on mice, in which the IEI distribution was relatively flat from 10 s upward (Silvani et al., 2015).

### Iron and Hemoglobin Assays

Hemoglobin concentration was measured from whole blood samples with a colorimetric assay (MAK115, Sigma-Aldrich; linear detection range: 0.9–200 mg/dL) performed in quadruplicate following manufacturer’s instructions. Non-heme iron was measured in the whole brainstem, liver, and serum with a published colorimetric assay protocol (Rebouche et al., 2004). Brainstem and liver homogenates were prepared by sonication in 10:1 (volume/weight) ultrapure water, added to an equal volume of protein precipitation solution (HCl 1 M and 10% TCA in ultrapure water), incubated at 95°C for 1 h to digest tissue proteins, cooled at room temperature for 2 min, mixed again, and centrifuged at room temperature at 8200 g for 10 min. Aliquots of 250 μL supernatant were combined with an equal volume of chromogen solution (ferrozine 0.508 mM, sodium acetate 1.5 M, and 1% v/v thioglycolic acid in ultrapure water). Dilute liver samples were also analyzed by mixing 125 μL supernatant with 62.5 μL of ultrapure water, 62.5 μL of protein precipitation solution, and 250 μL of working chromogen solution, in case the assayed iron content of the undiluted samples was beyond the upper limit (10 μg/mL) of the standard iron concentration curve (cf. below). For serum measurements, 100 μL serum were added to 100 μL ultrapure water and 250 μL of chromogen solution. Samples were prepared in duplicate, each with its own blank made by 250 μL of tissue extract supernatant and 250 μL of 1.5 M sodium acetate and 1% v/v thioglycolic acid in ultrapure water, without the addition of ferrozine. After 30 min incubation at room temperature, absorbance was measured in semi-micro cuvettes at a wavelength of 562 nm. Standard concentration/absorbance curves with iron concentrations of 0, 1, 2, 4, 6, 8, and 10 μg/mL (tissue samples) or 0, 0.5, 1, 1.5, 2, and 2.5 μg/mL (serum samples) were prepared fresh by diluting different volumes of a 100 μg/mL iron standard in ultrapure water (Titrisol Standard 1 g/100 mL, diluted 1% v/v in HCl 15% in high-purity water). Readings were performed in duplicate. The intra-assay coefficient of variation determined in preliminary analyses in quintuplicate was 0.9%.

### Statistical Analysis

Data were analyzed using SPSS Statistics (IBM Corp., Armonk, NY, United States) with mixed model analysis of variance (ANOVA) with Huynh-Feldt correction in case of failure of the sphericity assumption, and *t*-tests, with significance at *P* < 0.05. Data were shown as means ± SEM with *n* = 8 per dietary group for the analysis of TA EMG events, and with *n* = 11 per dietary group for the analysis of sleep. The sample size was set *a priori* with a statistical power analysis performed with G^∗^power software^1^. Calculations based on mean values and variances of published data on healthy mice (Silvani et al., 2015) indicated that a sample size of *n* = 8 per dietary group allowed a 80 and 95% statistical power to detect a 50% difference in the occurrence rate of TA-EMG events during non-REM sleep employing independent-samples and repeated-samples *t*-tests, respectively. The sample size for comparisons involving sleep variables was further increased to *n* = 11 per group due to the inclusion of three more mice per dietary group, in which one or both TA EMG signals were missing because of EMG electrode failure.

## Results

**FIGURE 1:**
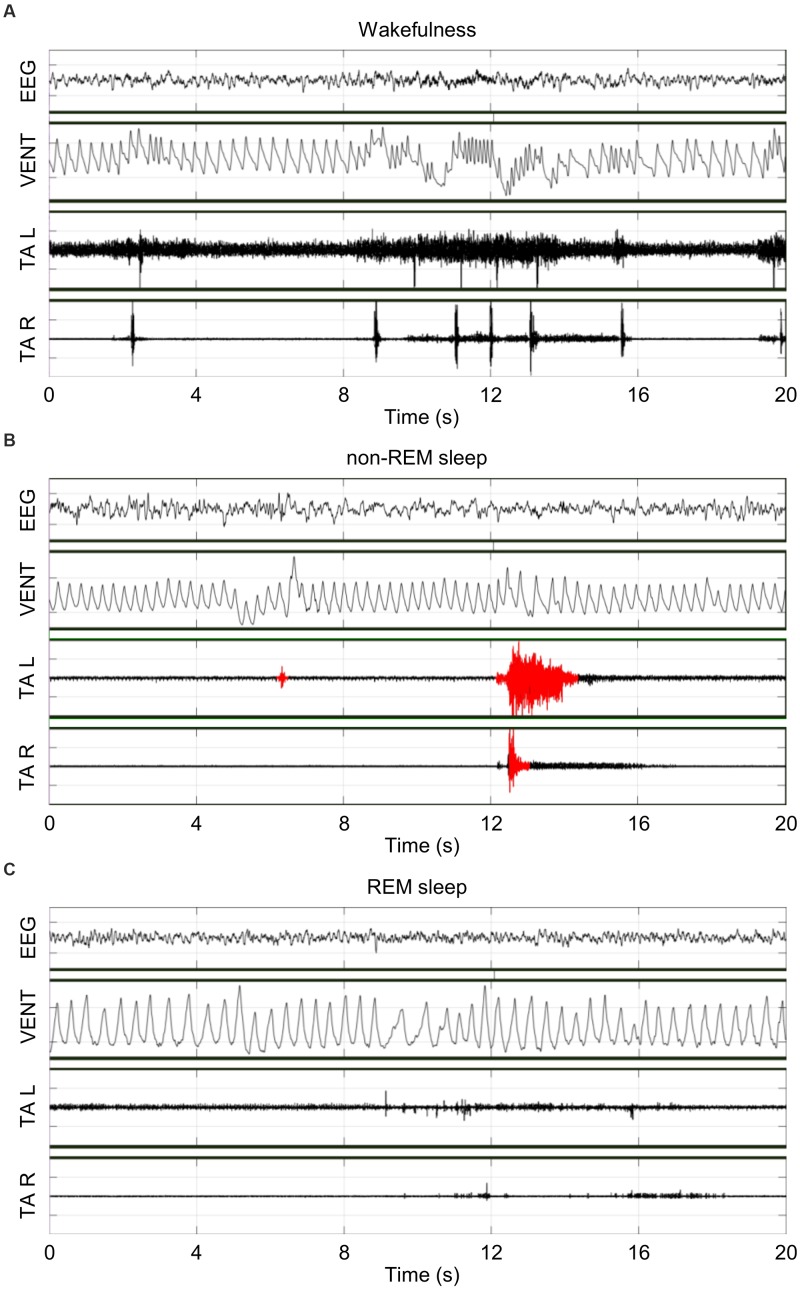
Representative raw tracings of the electroencephalogram (EEG), the raw ventilation signal yielded by the whole-body plethysmograph (VENT), and the left (L) and right (R) electromyogram (EMG) of tibialis anterior (TA) muscles during wakefulness **(A)**, non-rapid-eye-movement (non-REM) sleep **(B)**, and rapid-eye-movement (REM) sleep **(C)** in the same iron-deficient mouse in conditions of hypoxia. Scoring of TA EMG bursts (red) was performed only in non-REM sleep.

Figure 1 shows representative raw tracings of EEG, bilateral TA EMG, and breathing obtained inside a whole-body plethysmograph during wakefulness (A), non-REM sleep (B) and REM sleep (C). During the recordings, Ctrl and ID mice weighted 19.3 ± 0.4 g and 20.1 ± 0.5 g, respectively, with a loss of 8 ± 2% and 6 ± 1%, respectively, of the body weight at surgery (*P* = 0.366, *t*-test).

**FIGURE 2:**
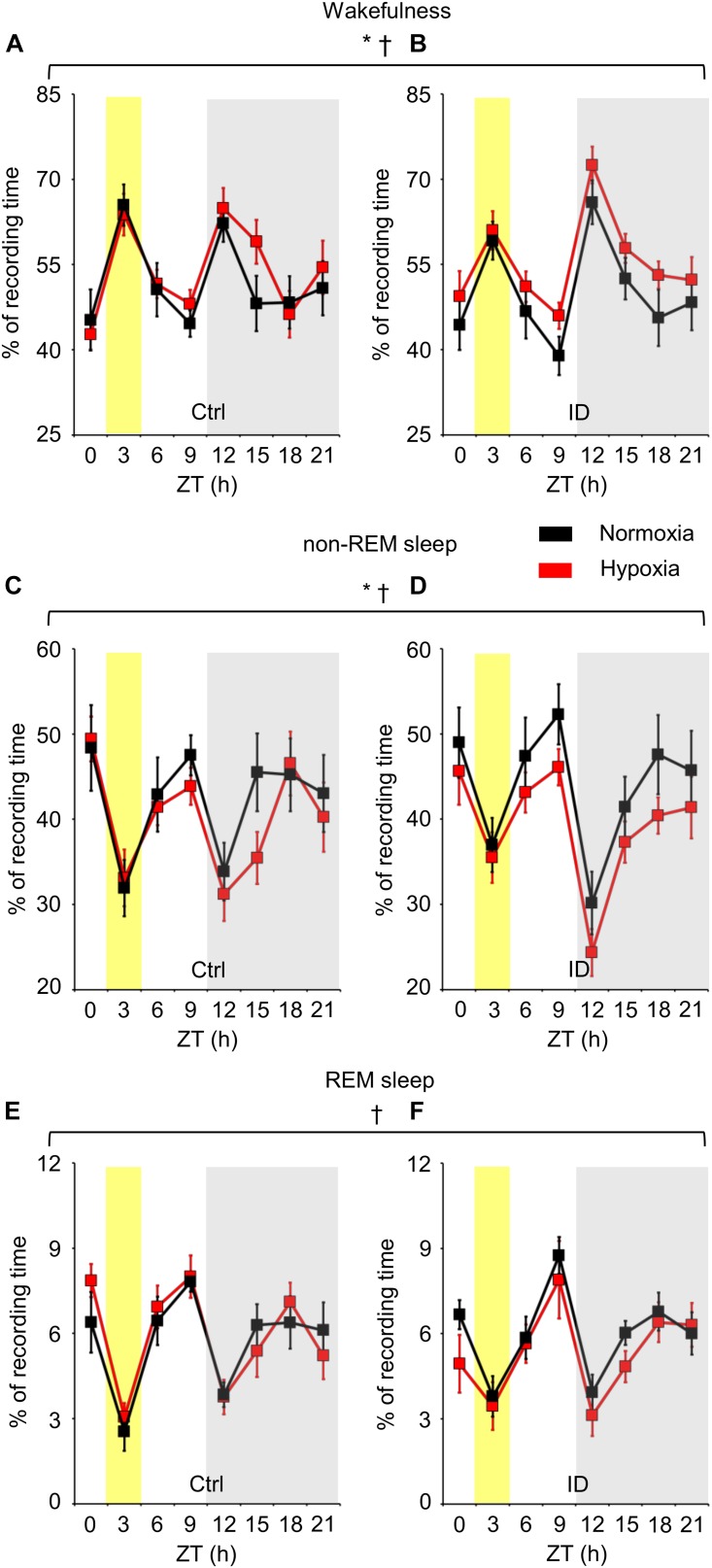
Percentages of recording time spent in wakefulness **(A,B)**, non-rapid-eye-movement (non-REM) sleep **(C,D)**, and rapid-eye-movement (REM) sleep **(E,F)**, averaged every 3 h in mice fed control diet (Ctrl) or iron-deficient diet (ID) during normoxia (black) and hypoxia (red). Zeitgeber time (ZT) 0 is time at lights on. The gray shading indicates the period with lights off (dark period). The yellow shading indicates the 3-h bin including ZT 3, when mice were first transferred to the recording environment. Data are means ± SEM with *n* = 11 per group. ^∗^ and ^†^, *P* < 0.05, main effects of hypoxia and ZT, respectively (ANOVA).

The time spent in wakefulness, non-REM sleep, and REM sleep is shown in Figure 2 in terms of percentages of total recording time as a function of hypoxia/normoxia, low/normal dietary iron, and ZT. As expected, the time spent in each wake-sleep state significantly depended on ZT (ANOVA main effect, *P* < 0.001). In particular, mice spent more time in wakefulness and less time in non-REM sleep and REM sleep during the dark period than during the light period (*P* < 0.001, *t*-test), exception made for the 3-h time bin of the light period around ZT 3. This corresponded to the start of the recordings, when mice were transferred from the dummy plethysmograph employed for habituation to the true plethysmograph. ANOVA main effects indicated that low dietary iron did not significantly affect the time spent in any wake-sleep state either in terms of ANOVA main effects (*P* ≥ 0.708) or of ANOVA interaction with other factors (*P* ≥ 0.214). On the other hand, regardless of iron deficiency, hypoxia significantly increased wakefulness time (*P* = 0.030) and decreased non-REM sleep time (*P* = 0.034), whereas it did not modify significantly REM sleep time (*P* = 0.483). These effects of hypoxia occurred irrespective of the light/dark period (*P* ≥ 0.720).

**FIGURE 3:**
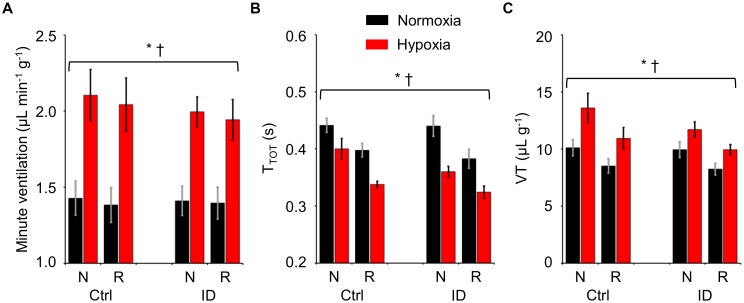
Values of minute ventilation per unit body weight **(A)**, ventilatory period (T_TOT_, **B**), and tidal volume per unit body weight (VT, **C**) during non-rapid-eye-movement sleep (N) and rapid-eye-movement sleep (R) in mice fed control diet (Ctrl) or iron-deficient diet (ID) during normoxia (black) and hypoxia (red). Data are means ± SEM with *n* = 11 per group. ^∗^ and ^†^, *P* < 0.05, main effects of hypoxia and sleep state, respectively (ANOVA).

The values of T_TOT_, VT per gram body weight, and minute ventilation per gram body weight are shown in Figure 3 as a function of hypoxia/normoxia, low/normal dietary iron, and sleep state. With respect to non-REM sleep, REM sleep entailed significant decreases both in T_TOT_ and in VT, which resulted in a slight but significant decrease in minute ventilation. Hypoxia significantly increased minute ventilation by decreasing T_TOT_ and by increasing VT (ANOVA main effects: hypoxia, *P* ≤ 0.006; sleep state, *P* ≤ 0.031; iron deficiency, *P* ≥ 0.269; ANOVA interactions, *P* ≥ 0.053).

**FIGURE 4:**
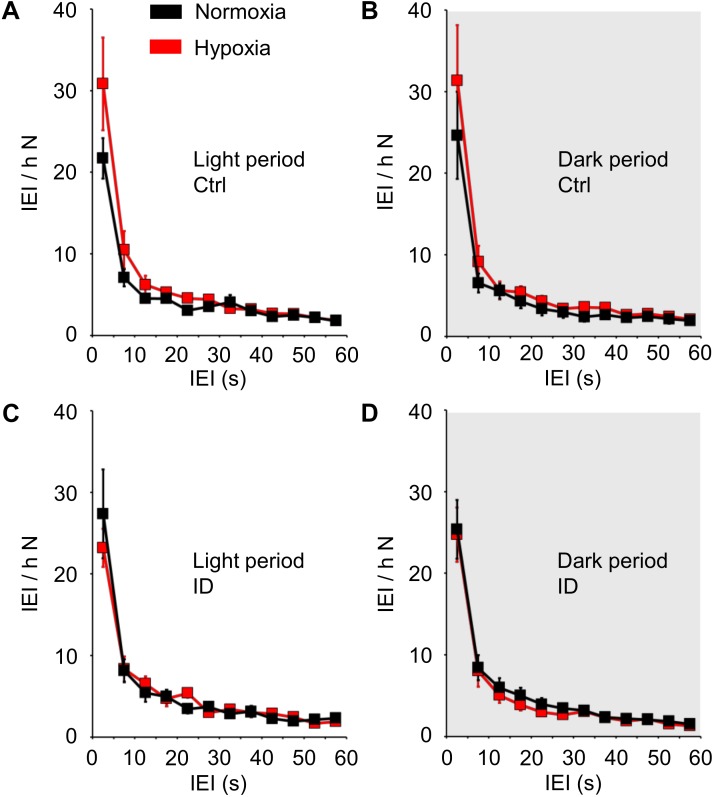
Distribution of the time intervals (inter-event intervals, IEI) between consecutive tibialis anterior (TA) electromyographic (EMG) events, expressed as number of IEI per hour of time spent in non-rapid-eye-movement sleep (N), in mice fed control diet (Ctrl, **A,B**) or iron-deficient diet (ID, **C,D**) during normoxia (black) and hypoxia (red). Data are shown separately for the light period **(A,C)** and dark period (gray shading, **B,D**) as means ± SEM with *n* = 8 per group. ANOVA did not indicate any significant effects of the light/dark period, hypoxia/normoxia, and Ctrl/ID group (cf. text for details).

The IEI distribution during non-REM sleep is shown in Figure 4 as a function of hypoxia/normoxia, low/normal dietary iron, and the light/dark period. The IEI distribution showed a prominent peak for IEI ≤10 s, followed by a longer tail up to IEI = 60 s. Hypoxia/normoxia, dietary iron, and light/dark period did not exert any significant effect on the IEI distribution during non-REM sleep, either individually or in combination with other factors (ANOVA: main effects, *P* ≥ 0.289; interactions, *P* ≥ 0.100). Neither hypoxia nor iron deficiency modified the general shape of the IEI distribution curve, and in particular no peak appeared in the range of long IEI >10 s.

**FIGURE 5:**
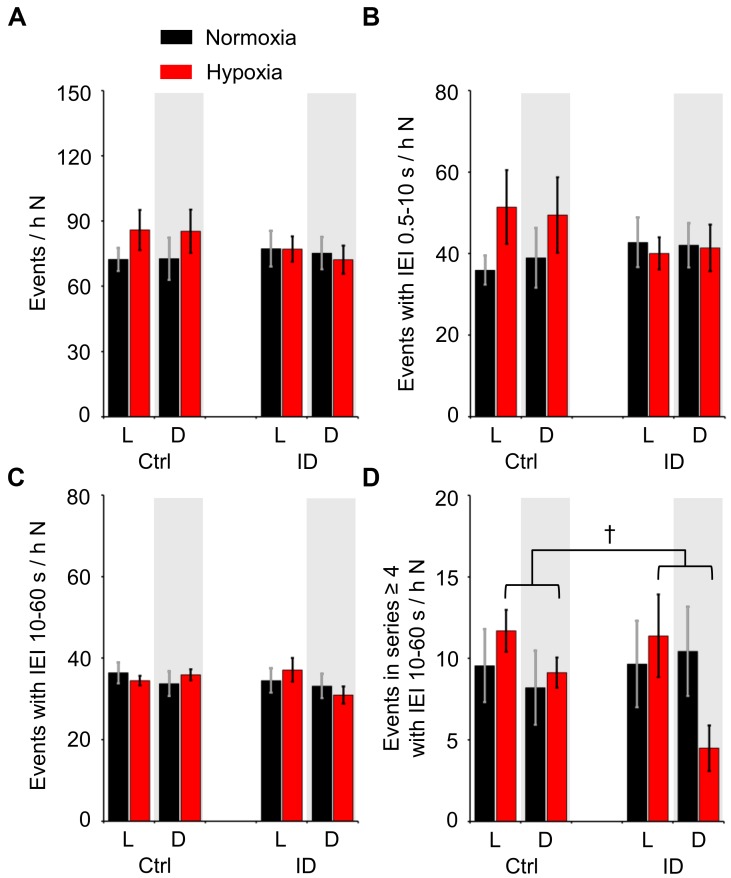
Number of different categories of tibialis anterior (TA) electromyographic (EMG) events per hour of time spent in non-rapid-eye-movement sleep (N) during the light period (L) and the dark period (D, gray shading) in mice fed control diet (Ctrl) or iron-deficient diet (ID). **(A)** Total number of TA EMG events per hour N time; **(B,C)** number of TA EMG events separated by inter-event intervals (IEI) between 0.5 and 10 s (short-IEI TA EMG events) or between 10 and 60 s (long-IEI TA EMG events), respectively, per hour N time; **(D)** number of long-IEI TA EMG events in series of four or more, expressed per hour N time. Data are means ± SEM with *n* = 8 per group. ^†^*P* < 0.05, L vs. D (*t*-test).

Figure 5 shows the total occurrence rate of TA EMG events during non-REM sleep, considering all TA EMG events together (panel A) or, separately, all the TA EMG events that had IEI ≤10 s (short-IEI TA EMG events, panel B), all the TA EMG events that had IEI between 10 and 60 s (long-IEI TA EMG events, panel C), and the long-IEI TA EMG events included in series of four or more events (“periodic” TA EMG events, panel D). The occurrence rate of all TA EMG events (Figure 5A) and that of short-IEI TA EMG events during non-REM sleep (Figure 5B) did not differ significantly as a function of hypoxia/normoxia, dietary iron, and the light/dark period (ANOVA: main effects, *P* ≥ 0.234; interactions, *P* ≥ 0.128). Conversely, the occurrence rate of long-IEI TA EMG events during non-REM sleep (Figure 5C) depended significantly on the 3-way interaction among hypoxia/normoxia, low/normal dietary iron, and the light/dark period (ANOVA, *P* = 0.017). However, none of the simple effects of the light/dark period, hypoxia, and iron deficiency on the occurrence of long-IEI TA EMG events during non-REM sleep was robust enough to reach statistical significance (*P* ≥ 0.368, *P* ≥ 0.068, and *P* ≥ 0.058, respectively; *t*-test). On the other hand, the occurrence rate of “periodic” TA EMG events during non-REM sleep (Figure 5D) significantly depended on the light/dark period and on its interaction with hypoxia/normoxia (ANOVA; *P* = 0.026 and *P* = 0.019, respectively). In particular, the occurrence rate of “periodic” TA EMG events was higher during the light than during the dark period in conditions of hypoxia, but not in conditions of normoxia (*P* = 0.010 and *P* = 0.780, respectively, *t*-test). The impact of low/normal dietary iron on “periodic” TA EMG events was not significant, either in terms of its main effect or of its interactions with other factors (ANOVA, *P* = 0.771 and *P* ≥ 0.076, respectively).

**FIGURE 6:**
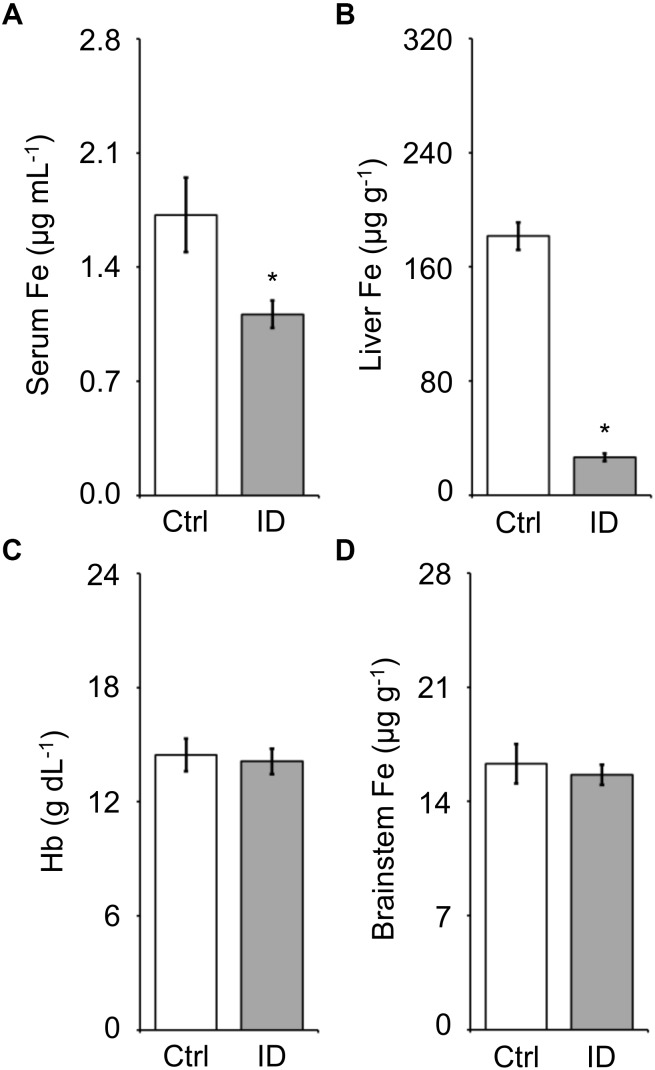
Values of iron (Fe) concentration measured in serum **(A)**, liver **(B)**, and brainstem **(D)** and of blood hemoglobin concentration **(C)** in the mice fed control diet (Ctrl) or iron-deficient diet (ID). Data are means ± SEM with *n* = 8–11 per group. ^∗^*P* < 0.05 vs. Ctrl (*t*-test).

The iron concentration in serum, liver, and brainstem and the blood hemoglobin concentration measured on samples harvested at the termination of the recordings after hypoxia (cf. methods) are reported in Figure 6. The low-iron diet caused a significant 38% decrease in serum iron and 85% decrease in liver iron stores (*P* = 0.022 and *P* < 0.001, respectively; *t*-test). Conversely, blood hemoglobin concentration and brainstem iron concentration were not significantly affected by the low-iron diet (*P* > 0.650, *t*-test).

## Discussion

We investigated whether female C57BL/6J mice exposed to mild hypoxia, alone or in combination with non-anemic iron deficiency, showed signs consistent with human RLS. We found that mild hypoxia was sufficient to elicit a significant increase in minute ventilation during sleep, and that 6 months of low-iron dietary treatment from weaning were sufficient to decrease serum and liver iron but not blood hemoglobin and brainstem iron storage. Mild hypoxia, either alone or in combination with non-anemic iron deficiency, caused a significant decrease in non-REM sleep time, irrespectively of the light/dark period. Although mild hypoxia tended to reorganize the distribution of sleep-related “periodic” TA EMG events during non-REM sleep, this was not accompanied by any robust increase in phasic TA EMG activity or by any significant change in the shape of the IEI distribution.

In our experiments, the decrease in the O_2_ fraction from 21 to 15% with a barometric pressure of 760 mm Hg entailed a decrease in the O_2_ partial pressure (PO_2_) from 160 to 114 mm Hg. A similar PO_2_ decrease is caused by the decrease in barometric pressure at approximately 2450 m above sea level. A study in a Turkish population found higher RLS prevalence in people living at higher altitudes, taking into account only locations up to 1800 m (Sevim et al., 2003). Another study on native South Americans in Ecuador found higher RLS prevalence in people living at 2816 m altitude than in people living at sea level (Castillo et al., 2006). A recent study on people living in the Himalayan and sub-Himalayan regions of India found an increased RLS prevalence in people living at 1900–2000 m than in those living at 400 m altitude, with no further increase in people living at 3200 m above sea level (Gupta et al., 2017). Taken together, these considerations indicate that the O_2_ fraction of the gas mixture breathed by mice in the conditions of mild hypoxia of the present study was in line with the PO_2_ in different high-altitude locations around the world with increased RLS prevalence (Sevim et al., 2003; Castillo et al., 2006; Gupta et al., 2017).

Previous experiments performed on male mice with genetic background and age that were similar, but not identical to those in our study (hybrid 129/Sv-C57BL/6 mice backcrossed to C57BL/6 for 5 generations, aged 24–36 weeks vs. C57BL/6J mice aged 27–28 weeks) did not show significant changes in sleep time or in tidal volume or ventilatory rate during sleep when mice breathed a 15% O_2_ hypoxic mixture for 6 h (Nakamura et al., 2007). The discrepancy with our results may be because of differences in the sex of the mice or the duration of hypoxia or because of slight differences in genetic background and age of the mice.

A decrease in non-REM sleep time during the night has been reported in patients with RLS, together with a decrease in sleep efficiency and increases in sleep fragmentation and arousal index, and also with a decrease in REM sleep time (Hornyak et al., 2007). RLS symptoms have a circadian rhythm of prevalence during the evening/night (Hening et al., 1999; Michaud et al., 2005), which corresponds to the beginning of the light period in mice. On these bases, we reasoned that a reduction in sleep time restricted to or most evident during the light (rest) period in our mice would be most consistent with RLS signs in humans. However, contrary to our hypothesis, the decrease in non-REM sleep time we found in mice during mild hypoxia occurred during the light and dark periods irrespective of ZT (Figure 2).

More than 80% of patients with RLS show more than 5 PLMS per hour of sleep (Montplaisir et al., 1997). The most recent guidelines of the World Association of Sleep Medicine (WASM) recommend scoring PLMS as phasic TA EMG events that comply with a set of criteria that include the length of inter-movement intervals (10–90 s) and the number of events in a series (4 or more) (Ferri et al., 2016). Recent data suggest decreasing the upper limit of the inter-movement interval of PLMS to 60 s (Ferri et al., 2018). Adult patients with RLS typically show an increased occurrence rate not only of PLMS, but also of all leg movements during sleep (LMS) compared to control subjects (Ferri et al., 2017a). The short-interval LMS with inter-movement interval between 0.5 and 10 s entail greater accompanying changes in heart rate compared with PLMS (Ferri et al., 2017b). Short-interval LMS also occur in healthy human subjects, albeit at a much lower rate than in patients with RLS (Ferri et al., 2017a), as well as in healthy rats and mice (Silvani et al., 2015). In this study, we confirmed that the distribution of TA EMG events during non-REM sleep in mice had a prominent peak for IEI <10 s, corresponding to short-interval LMS, but no other peak for IEI between 10 and 60 s, which would be consistent with human PLMS (Figure 4). This distribution was remarkably stable across the 24-h, without significant differences between the light period and the dark period. Little is known on the circadian rhythm of LMS in human subjects. Limited evidence on four subjects with a high occurrence rate of PLMS but no RLS suggested a circadian rhythm of PLMS occurrence, which peaked at the transition between subjective day and night (Duffy et al., 2011). It is unclear whether this also holds for patients with RLS (Michaud et al., 2005) and/or for other categories of LMS, such as the short-interval LMS. It is known, however, that PLMS occurrence peaks during the second hour of sleep and then progressively declines throughout the night in patients with RLS (Ferri et al., 2017a), whereas the occurrence of short-interval LMS is stable throughout the night (Silvani et al., 2015; Ferri et al., 2017a).

Despite the absence of a clear-cut peak of the distribution of TA EMG events during non-REM sleep for IEI >10 s, some TA EMG events during non-REM sleep satisfied sequence and IEI criteria similar to those for human PLMS (cf. methods). We found that the time structure of these “periodic” TA EMG events was significantly affected by hypoxia, which enhanced a nychthemeral rhythm with the higher occurrence rate during the light (rest) period (Figure 5). Nonetheless, the occurrence rate of these “periodic” TA EMG events during non-REM sleep was not significantly higher in conditions of hypoxia than in those of normoxia either in the light or in the dark period. Similarly, hypoxia did not significantly increase the occurrence during non-REM sleep of long-interval (10–60 s) TA EMG events of any sequence length, of short-interval (<10 s) TA EMG events, or of all TA EMG events regardless of their IEI (Figure 5). Clearly, these findings are not consistent with the marked differences in LMS occurrence between adult patients with RLS and control subjects (Ferri et al., 2017a). Thus, they do not support the hypothesis that hypoxia increases the occurrence of TA EMG events during non-REM sleep in mice. It is worth remarking that an *a priori* statistical power analysis estimated an extremely high (95%) power to detect even a modest 50% difference in the occurrence rate of TA EMG events during non-REM sleep due to hypoxia (cf. methods).

In our study, long-term administration of a low-iron diet to female C57BL/6J mice for 6 months from weaning resulted in a dramatic 85% depletion of iron stores in the liver, which is the main iron storage organ (Ganz, 2013), and in a 38% decrease in serum iron concentration, but did not result in significant decreases in hemoglobin concentration and brainstem iron levels (Figure 6). This corresponds to non-anemic iron deficiency, a condition of iron-deficient erythropoiesis (Adamson, 2008). This condition did not lead *per se* to any significant change in sleep (Figure 2) or TA EMG events during sleep (Figures 4, 5). Again, it is worth remarking that our statistical power analysis estimated a 80% power to detect a 50% difference in TA EMG events due to iron deficiency (cf. methods).

Employing the same low-iron diet as in the present study but for a shorter period of time (15 weeks vs. 24 weeks), male C57BL/6J mice were previously reported to develop a 33% decrease in serum iron concentration, a shift in the light/dark rhythm of motor activity, with increased voluntary activity (wheel running) at the end of the active (dark) period, and increased pain responses, which were claimed to resemble features of patients with RLS (Dowling et al., 2011). A similar alteration in light/dark rhythms of non-REM sleep had been previously reported on male C57BL/6J mice administered a diet with a slightly lower iron content (6.6 ppm iron) for a much shorter period of time (6 weeks) (Dean et al., 2006). Neither of these previous studies (Dean et al., 2006; Dowling et al., 2011) measured hemoglobin concentration, body iron stores, or brain iron concentration, which would have helped clarify the reasons for the discrepancy with our results. On the other hand, both studies (Dean et al., 2006; Dowling et al., 2011) involved male mice, whereas ours involved female mice because of evidence that RLS is more prevalent in females (Manconi et al., 2012). However, we studied virgin female mice to decrease experimental variability due to differences in litter size, whereas previous pregnancies are known to contribute to gender differences in RLS prevalence (Pantaleo et al., 2010). It is therefore possible that male mice are more susceptible than virgin female mice to the sleep and motor consequences of iron deficiency. Regardless, the face validity for RLS of increased motor activity (Dowling et al., 2011) and decreased non-REM sleep (Dean et al., 2006) at the end of the active period may be questioned, given that that, as previously remarked, the circadian rhythm of symptoms in patients with RLS prominently involves the rest period of the day (Hening et al., 1999; Michaud et al., 2005). A recent study on iron-deficient rats, which developed a dramatic, approx. 50% decrease in hematocrit, and a massive, 600–700% increase in PLMS occurrence rate, also found a peak in the occurrence rate of PLMS and periodic leg movements during wakefulness (PLMW) in the last hours of the active period (Lai et al., 2018), but no decrease in sleep during the last hours of the active period (Lai et al., 2017). Sleep time during the active period was actually *increased* in some of the rats (Lai et al., 2018), which may translate excessive daytime sleepiness occasionally reported in patients with RLS (Fulda and Wetter, 2007; Kallweit et al., 2009). However, it is actually the *lack* of profound sleepiness during daytime in spite of poor nocturnal sleep that is considered a characteristic finding in most patients with RLS, and is therefore included as a supporting clinical features for RLS diagnosis in the updated IRLSSG consensus criteria (Allen et al., 2014). These differences in the interpretation of experimental results highlight the lack of consensus on the optimal experimental readouts for RLS in animal models, given that RLS is diagnosed entirely based on the subjective symptoms (Allen et al., 2017).

Taken together, our results do not support the hypothesis that mild hypoxia is sufficient to elicit the set of explicit, objective experimental RLS readouts that we employed in this study on mice. Based on the previous considerations, it is possible that the objective metrics used in the present study did not capture the entire spectrum of clinical symptoms that lead to diagnosis of RLS. Nonetheless, having explicit, objective readouts is unavoidable for the use of animal models to study RLS.

A limitation of our study design is that for logistical reasons, we did not study mice raised under hypoxic conditions. This might explain the discrepancy between our negative findings and the clinical association between RLS and chronically hypoxic conditions (Sevim et al., 2003; Castillo et al., 2006; Kapsimalis and Kryger, 2009; Lo Coco et al., 2009; Gupta et al., 2017). Nonetheless, our study involved mice exposed to mild hypoxia for a total of 48 h. This time is relatively long compared to that employed in other studies on sleep physiology in mice (Nakamura et al., 2007) and sufficient to entail changes in brain mitochondrial biogenesis (Gutsaeva et al., 2008) and brain HIF expression (Shao et al., 2005), which are thought to underlie causal links among hypoxia, iron deficiency, and RLS (Connor et al., 2017).

While it remains conceivable that mild hypoxia requires additional promoting factors to elicit RLS signs, our data do not support the hypothesis that non-anemic iron deficiency is one of these factors. We cannot exclude that more severe levels of hypoxia alone or in combination with severe, anemic iron deficiency, may elicit RLS signs in mice. However, these severe conditions arguably characterize only a small fraction of the patients with RLS. We also cannot exclude that mild hypoxia is effective in eliciting RLS signs if iron deficiency involves the neural tissue. Neural iron deficiency did not occur in our study (Figure 6), but has been consistently associated with human RLS (Trenkwalder et al., 2016). We studied mice of the C57BL/6J strain, which is reportedly the most widely used inbred strain^2^. In a study of brain iron concentration performed on a panel of 24 inbred mouse strains under a low dietary iron regimen, C57BL/6J mice ranked 12/24 and 20/24 in terms of decreasing iron concentration in the ventral midbrain and caudate/putamen, respectively, indicating a median to high susceptibility of C57BL/6J mice to brain iron deficiency depending on the brain region (Jellen et al., 2012). Studying mice at the high end of the genetic spectrum of susceptibility to brain iron deficiency may be a promising strategy for future experiments. Nonetheless, the distribution of brain iron estimates often shows a wide overlap between individuals with and without RLS (Allen et al., 2001; Rizzo et al., 2013; Li et al., 2016).

The mechanisms responsible for the differential susceptibility to RLS in subjects with similar levels of brain iron still remain unclear. Genetic factors may be at stake, as a recent meta-analysis of genome-wide association studies highlighted 19 genetic risk loci for RLS, mainly related to neurodevelopment (Schormair et al., 2017). Interestingly, at least two of the genes that have been implicated -BTBD9 (DeAndrade et al., 2012) and MEIS1 (Salminen et al., 2018)-have already been mutated in mice, resulting in alterations of sleep and motor activity that may be relevant for RLS pathophysiology.

## Conclusion

In conclusion, our results did not support the hypothesis that mild hypoxia, alone or in combination with non-anemic iron deficiency, is sufficient to elicit signs consistent with human RLS. Other susceptibility factors not modeled in the present study, such as brain iron deficiency or specific genetic risk factors, may be key for the development of RLS.

## Author Contributions

AS, MM, and GZ designed the study. VLM, SB, SA, CB, and AV contributed to surgery and recordings. SB contributed to sleep scoring. VLM contributed to tibialis anterior electromyographic event scoring. VLM, SA, AV, SB, CB, and AS contributed to iron and hemoglobin assays. AS contributed to data analysis and statistics. AS drafted the manuscript. All authors reviewed the manuscript for important intellectual content.

## Conflict of Interest Statement

The authors declare that the research was conducted in the absence of any commercial or financial relationships that could be construed as a potential conflict of interest.

## Footnotes

1 www.gpower.hhu.de

2 www.jax.org
